# Association between loneliness in childhood and first-episode psychosis

**DOI:** 10.1192/j.eurpsy.2024.225

**Published:** 2024-08-27

**Authors:** C. M. Díaz-Caneja, L. Donaire, V. Cavone, Á. Andreu-Bernabeu, J. González-Peñas, M. Díaz-Marsá, R. Rodríguez-Jiménez, Á. Ibáñez, E. Baca-García, J. C. Leza, M. F. Bravo-Ortiz, J. L. Ayuso-Mateos, C. Arango

**Affiliations:** ^1^Department of Child and Adolescent Psychiatry, Institute of Psychiatry and Mental Health, Hospital General Universitario Gregorio Marañón, IiSGM, CIBERSAM, ISCIII, School of Medicine, Universidad Complutense; ^2^Department of Child and Adolescent Psychiatry, Institute of Psychiatry and Mental Health, Hospital General Universitario Gregorio Marañón, IiSGM, CIBERSAM, School of Medicine, Universidad Complutense; ^3^Department of Psychiatry, Instituto de Investigación Hospital Clínico San Carlos (IdISSC), CIBERSAM, ISCIII, School of Medicine, Universidad Complutense; ^4^Department of Psychiatry, Instituto de Investigación Sanitaria Hospital 12 de Octubre (imas 12), CIBERSAM, ISCIII, School of Medicine, Universidad Complutense; ^5^Department of Psychiatry, Hospital Universitario Ramón y Cajal, IRYCIS, CIBERSAM, ISCIII, Universidad de Alcalá; ^6^Department of Psychiatry, Hospital Fundación Jiménez Díaz, Hospital Rey Juan Carlos, Hospital General de Villalba, Hospital Infanta Elena, Instituto de Investigación Sanitaria Fundación Jiménez Díaz, CIBERSAM, ISCIII, Universidad Autónoma de Madrid, Madrid, Spain; ^7^Universidad Catolica del Maule, Talca, Chile; ^8^Department of Psychiatry, Centre Hospitalier Universitaire, Nîmes, France; ^9^Department of Pharmacology & Toxicology, School of Medicine, Universidad Complutense de Madrid, Instituto de Investigación Sanitaria Hospital 12 de Octubre (imas 12), IUIN; ^10^Department of Psychiatry, Clinical Psychology and Mental Health, Instituto de Investigación Hospital Universitario La Paz, IdiPaz, Hospital Universitario La Paz, CIBERSAM, ISCIII, Department of Psychiatry, Universidad Autónoma de Madrid; ^11^Department of Psychiatry, Hospital Universitario de La Princesa, Instituto de Investigación Sanitaria del Hospital Universitario de La Princesa, IIS Princesa, CIBERSAM, ISCIII, School of Medicine, Universidad Autónoma de Madrid, Madrid, Spain

## Abstract

**Introduction:**

Evidence from observational and genetic studies suggests a bidirectional relationship between loneliness and psychosis. To our knowledge, no previous study has assessed the association between loneliness in childhood and first-episode psychosis (FEP).

**Objectives:**

We aimed to assess the association between loneliness in childhood and the odds of FEP and clinical variables of interest (i.e., diagnosis and clinical and functional severity) in FEP and to explore gender differences in this association.

**Methods:**

This was an observational, case-control study, based on the AGES-CM cohort, a longitudinal prospective study including patients with FEP ages 7-40, their first-degree relatives, and an age- and sex-matched sample of controls in seven university hospitals in the region of Madrid. We assessed loneliness in childhood with the question *“Have you ever felt lonely for more than 6 months before the age of 12”* and objetive social isolation with the peer relationships item from the childhood subscale of the Premorbid Adjustment Scale. We conducted logistic and linear regression analyses to assess the association between childhood loneliness and i) the odds of presenting a FEP and ii) clinical variables of interest (diagnosis and scores on positive, negative, general, depressive, and manic symptoms and functioning), while adjusting for demographic variables.

**Results:**

The study sample comprised 285 patients with FEP (32.6% female, age 24.50 ± 6.2 years) and 546 controls (48.7% female, age 25.93 ± 5.5 years). Loneliness in childhood was associated with increased odds of FEP (adjusted odds ratio; aOR: 2.17, 95% CI [1.40-3.51], p=.002). This association remained significant after controlling for objective social isolation in childhood (aOR:2.70, IC 95% [1.58-4.62], p<.001).

The effect of the association was stronger in females (aOR:4.74, 95% CI [2.23-10.05], p<.001) than in males (aOR:1.17, IC 95% [0.63-2.19], p=.623). In females with FEP, loneliness in childhood was significantly associated with increased odds of receiving a diagnosis of other psychosis (aOR:0.155, 95% CI [0.048-0.506], p=.002) relative to an SSD diagnosis. In the FEP sample, loneliness in childhood was associated with greater severity of positive and affective symptoms and worse functioning.

**Conclusions:**

Loneliness in childhood is associated with increased odds of FEP and clinical variables of interest. This suggests the potential role of this phenotype as an early risk marker for psychosis that could help guide targeted interventions.

**Disclosure of Interest:**

C. Díaz-Caneja Grant / Research support from: Instituto de Salud Carlos III (PI17/00481, PI20/00721, JR19/00024), European Union, Consultant of: Angelini, L. Donaire: None Declared, V. Cavone: None Declared, Á. Andreu-Bernabeu: None Declared, J. González-Peñas: None Declared, M. Díaz-Marsá: None Declared, R. Rodríguez-Jiménez: None Declared, Á. Ibáñez: None Declared, E. Baca-García: None Declared, J. C. Leza: None Declared, M. F. Bravo-Ortiz: None Declared, J. L. Ayuso-Mateos: None Declared, C. Arango Grant / Research support from: Madrid Regional Government (R&D activities in Biomedicine S2022/BMD-7216 AGES 3-CM), Instituto de Salud Carlos III, European Union, Consultant of: Acadia, Angelini, Biogen, Boehringer, Gedeon Richter, Janssen Cilag, Lundbeck, Medscape, Menarini, Minerva, Otsuka, Pfizer, Roche, Sage, Servier, Shire, Schering Plough, Sumitomo Dainippon Pharma, Sunovion and Takeda

